# Home high-flow therapy during recovery from severe chronic obstructive pulmonary disease (COPD) exacerbation: a mixed-methods feasibility randomised control trial

**DOI:** 10.1136/bmjresp-2024-002698

**Published:** 2025-01-06

**Authors:** Rebecca F D'Cruz, Anne Rossel, Georgios Kaltsakas, Eui-Sik Suh, Abdel Douiri, Louise Rose, Patrick B Murphy, Nicholas Hart

**Affiliations:** 1 Lane Fox Clinical Respiratory Physiology Research Unit, Guy's and St Thomas' NHS Foundation Trust, London, UK; 2 Centre for Human and Applied Physiological Sciences, King's College London, London, UK; 3 Division of General Internal Medicine, Geneva University Hospitals, Geneve, Switzerland; 4 Faculty of Life Sciences and Medicine, King's College London, London, UK; 5 School of Population Health and Environmental Sciences, King's College London, London, UK; 6 King's College London Florence Nightingale School of Nursing and Midwifery, London, UK; 7 Florence Nightingale Faculty of Nursing, Midwifery and Palliative Care, King's College London, London, UK

**Keywords:** COPD Exacerbations, Emphysema, Lung Physiology, Patient Outcome Assessment, Respiratory Function Test, Respiratory Muscles

## Abstract

**Introduction:**

Patients recovering from severe acute exacerbations of chronic obstructive pulmonary disease (AECOPD) have a 30-day readmission rate of 20%. This study evaluated the feasibility of conducting a randomised controlled trial to evaluate clinical, patient-reported and physiological effects of home high-flow therapy (HFT) in addition to usual medical therapy, in eucapnic patients recovering from AECOPD to support the design of a phase 3 trial.

**Methods:**

A mixed-methods feasibility randomised controlled trial (quantitative primacy, concurrently embedded qualitative evaluation) (ISRCTN15949009) recruiting consecutive non-obese patients hospitalised with AECOPD not requiring acute non-invasive ventilation. Participants were randomised to receive usual care or usual care and home HFT (37°C, 30 L/min) with weekly home-based follow-up for 4 weeks to collect data on: device usage, breathlessness (modified Borg scale, visual analogue scale, Multidimensional Dyspnoea Profile), health-related quality of life (COPD Assessment Test (CAT), Clinical COPD Questionnaire), pulse oximetry, spirometry and inspiratory capacity, parasternal electromyography and actigraphy. Semistructured interviews were conducted in week 4. Trial progression criteria were: ≥40% of eligible patients randomised, ≤20% attrition, ≥70% complete data, and no device-related serious adverse events (SAE).

**Results:**

18 of 45 eligible patients were randomised (age 69±5 years, 44% female, body mass index 23±5 kg/m^2^, forced expiratory volume in 1 second 32±12%). One withdrew following non-respiratory hospitalisation. Complete outcome measures were collected in >90% of home assessments. There were no device-related SAE. Daily HFT usage was 2.7±2.2 hours in week 1, falling to 2.3±1.4 hours by week 4. Temperature and flow settings were modified for comfort in 6 cases. Higher HFT usage was associated with lower symptom burden (CAT p=0.01). Interviews highlighted ease of device use, reduced salbutamol usage, and improved sputum production and clearance.

**Conclusions:**

The data from this feasibility study support the progression to a phase 3 randomised clinical trial investigating the effect of home (HFT) on admission-free survival in COPD patients recovering from a severe exacerbation.

**Trial registration number:**

The study received ethical approval (REC19/LO/0194) and was prospectively registered (ISRCTN15949009).

**WHAT IS ALREADY KNOWN ON THIS TOPIC**

- High-flow therapy (HFT) delivers heated humidified gas (air with or without oxygen enrichment) to the airways. Preliminary data in clinically stable patients with COPD and chronic respiratory failure indicates possible clinical benefits including reduced exacerbation rates and breathlessness and improved gas exchange and exercise capacity. Patients hospitalised with severe COPD exacerbation even without comorbid respiratory failure are at high-risk of re-exacerbation and readmission despite pharmacological optimisation and pulmonary rehabilitation.

**WHAT THIS STUDY ADDS**

- Home HFT is safe and acceptable to patients to patients recovering from severe COPD exacerbation, and it is feasible to conduct a phase 3 randomised clinical trial evaluating the effect of home HFT on admission-free survival.

**HOW THIS STUDY MIGHT AFFECT RESEARCH, PRACTICE OR POLICY**

- This study informs the design of a UK phase 3 clinical trial to investigate whether home HFT is an effective adjunct to reduce readmission and mortality in patients recovering from severe COPD exacerbation.

## Introduction

 Acute exacerbations of chronic obstructive pulmonary disease (COPD) are a common cause of admission to hospital in the UK and worldwide. Up to 24% of patients recovering from severe COPD exacerbation are readmitted to hospital within thirty days of discharge.^1^ This proportion has remained static despite implementation of readmission prevention strategies, including changes in process of care as well as optimisation of medical management.^2^

In patients who are hospitalised with COPD exacerbation, without evidence of persistent hypercapnia during recovery, interventions to reduce risk of re-exacerbation and readmission are limited. Although inhaled bronchodilation and mucolytics have been shown to reduce exacerbation risk^3 4^ and emerging data indicate that type 2 inflammation inhibition can reduce moderate and severe exacerbation rates,^5^ these studies have targeted stable COPD patients. In hospitalised eucapnic COPD patients, evidence supports non-pharmacological interventions, such as pulmonary rehabilitation, to reduce 12-month readmission and mortality risk.^6^ However, shortfalls in access, uptake and programme completion have failed to change clinical outcomes.^7^ Other interventions, such as home non-invasive ventilation (NIV) with oxygen therapy in post-acute COPD patients with persistent hypercapnia and long-term oxygen therapy (LTOT) in stable hypoxaemic COPD patients, have been shown to increase admission-free survival^8^,^11^ and are part of clinical guidelines.^12^

Home high-flow therapy (HFT) delivers heated, humidified air or air-oxygen blend, and there is growing evidence of clinical and physiological effects in COPD patients following acute treatment with mechanical ventilation^13^,^15^ and stable patients with hypoxaemia and hypercapnia.^16^,^18^ Short-term physiological studies in COPD patients demonstrate that HFT reduces respiratory rate, respiratory drive and inspiratory effort, by changing breathing pattern and increasing expiratory time, and reduces breathlessness.^19 20^ These observations of the effect of HFT during acute exacerbation recovery, combined with a physiological home monitoring study which demonstrated early post-discharge improvements in airflow obstruction, neural respiratory drive and breathlessness,^21^ provide the rationale to use HFT in eucapnic COPD patients in the recovery phase at home. Additionally, despite clinical trials demonstrating reduced exacerbation frequency and symptom burden with home HFT and LTOT, there has been no demonstrable impact on hospital admissions and readmissions, providing further support for the rationale to target eucapnic normoxaemic post-acute COPD patients.

The aim of this study was to determine the feasibility of conducting a randomised clinical trial to evaluate clinical, patient-reported and physiological effects of home HFT, in addition to usual medical therapy, in eucapnic patients recovering from severe COPD exacerbation following hospitalisation.

## Methods

This was a single-centre open label feasibility mixed-methods study comprising a parallel group randomised controlled trial (RCT) with embedded concurrent qualitative process evaluation reported using the feasibility trials Consolidated Standards of Reporting Trials statement and Consolidated criteria for Reporting Qualitative research checklist.^22 23^ The study received ethical approval (REC19/LO/0194) and was prospectively registered (ISRCTN15949009).

Consecutive patients presenting to the Accident and Emergency Department at a large urban university hospital in the UK who were hospitalised with physician-diagnosed COPD exacerbation were screened for eligibility. Inclusion criteria were: admission to hospital with an acute exacerbation of COPD, age 40–80 years, ≥10 pack year smoking history, body mass index ≤35 kg/m^2^, cognitively able to provide informed consent and adhere to the study protocol, and recruitment within 24-hours of admission. Patients with comorbidities likely to influence readmission risk (including severe heart failure, end stage renal failure, active malignancy), or those who were hypercapnic (arterial partial pressure of carbon dioxide (PaCO_2_) >7.0 kPa) during the index hospitalisation, required acute NIV or were established on domiciliary NIV, or continuous positive airway pressures were excluded.

Prior to discharge, patients were randomised 1:1 to receive usual medical care^24^ or usual medical care with home HFT (*myAIRVO2*, *Fisher & Paykel Healthcare, NZ*) with established readmission predictors as stratification factors: annual exacerbation frequency (≥2 or <2) and admission-to-discharge change in parasternal electromyography (EMG_para_) (>3.1% or ≤3.1%).^25 26^ Randomisation with a concealed allocation sequence was implemented (Online Randomisation Service v1.5.2, King’s Clinical Trials Unit). Strata were generated for each combination of covariates with subjects assigned to the appropriate stratum and then block randomisation to assign patients to a treatment group. Intervention arm participants underwent a period of inpatient HFT acclimatisation and training prior to hospital discharge, with settings titrated to comfort, aiming for temperature of 37°C and flow of 30 L/min. Participants were informed that benefits have been observed with home HFT usage of 6 hours per 24-hour period.^16^ A defined device prescription was not provided in this feasibility trial as one aim was to explore usage patterns.

### Quantitative data collection

Demographics, anthropometrics and clinical data (medical history, admission venous and arterial blood tests, chest radiograph findings, vital observations) were recorded at baseline inpatient assessments. The study team performed weekly home-based assessments for 4 weeks post-discharge to measure patient-reported and physiological outcomes and device usage. Health-related quality of life (HRQoL) and symptom burden were assessed using the COPD Assessment Test (CAT), Clinical COPD Questionnaire (CCQ) and Multidimensional Dyspnoea Profile.^27^,^29^ Physiological measures included vital observations, spirometry and inspiratory capacity (*EasyOne Diagnostic Spirometer, ndd Medical Technologies, Switzerland*), which were performed in accordance with international guidelines and reported methods.^21 30^ Neural respiratory drive was quantified using EMG_para_, using previously reported methods to derive values for mean EMG_para_, EMG normalised to maximal inspiratory manoeuvre (EMG_para%max_) and EMG as product of respiratory rate (neural respiratory drive index, NRDI).^21^ Patients recorded breathlessness daily (modified Borg scale (mBorg); visual analogue scale (VAS)) on a paper symptom diary. Physical activity and sleep quality were measured with a triaxial accelerometer (*Actiwatch Spectrum, Philips, USA*), worn continuously on the non-dominant wrist for the study duration. HFT usage was obtained directly from devices.

### Qualitative data collection

Embedded qualitative research, underpinned by the Theoretical Framework of Acceptability (TFA), was used to explore patients’ experiences of home HFT during COPD exacerbation recovery, including its acceptability and barriers and facilitators to its use.^31^ Purposive sampling of intervention arm participants to include patients with a range of characteristics (gender, ethnicity, living alone, smoking status) was conducted. One face-to-face semistructured interview was undertaken with an investigator trained in qualitative methodology (RFD). The topic guide was developed using data and expertise in experiences of NIV in COPD and was reviewed for face validity by another team member with expertise in qualitative research (LR).^32 33^ Interviews were conducted in patients’ homes to enable direct visualisation of the environment in which the device was used, thereby providing additional cues to factors influencing acceptability and to reduce interviewer-subject power imbalance.

To determine trial feasibility, *a priori* progression criteria were defined as: ≥40% of eligible patients consenting to randomisation and ≤20% attrition (based on our previous trial data comparing usual care with or without home non-invasive respiratory support in COPD patients recovering from severe exacerbation),^8^ ≥70% data collected for outcome measures to enable sample size calculation for the full-scale clinical trial, and no device-related SAE as a safety outcome for this novel application of a medical device.

### Statistical analysis

A sample size of 80 patients was planned to estimate feasibility (recruitment of 40% eligible subjects, 70% completion of outcome measures) and clinical (anticipated 20% 30-day readmission) outcomes with a 95% CI of ±10%.^34^ Quantitative data are reported descriptively (mean±SD, median (IQR), number (%)), using an intention-to-treat principle. Correlation analyses were performed using Pearson or Spearman Rank correlation (SPSS, v28, *IBM Corp, USA*). Qualitative data were analysed using framework analysis, involving familiarisation of transcripts, coding using TFA constructs and matrix formation (NVivo, v12, *QSR International, Australia*).^31 35^ Quantitative and qualitative integration was used to provide illustration of the data in a real-world context, with convergent validation (assumption of improved validity if the quantitative and qualitative findings agree) and analytic density (a wider and deeper picture of the research from multiple perspectives).^36 37^ Integration was achieved using adapted triangulation and convergence coding, whereby the findings of each study were sorted into meta-themes based on the outcome measures. Meta-themes were mapped into a matrix containing convergence coding for agreement, partial agreement, dissonance or silence. A convergence assessment was used to quantify the level of agreement, and a completeness comparison defined differences in the contributions of the datasets to the overall evaluation.^38^

### Patient and public involvement (PPI)

Patient members of the Lane Fox Clinical Respiratory Physiology Research PPI Group supported the design and conduct of this study. The patient information leaflet, trial protocol, study intervention, outcome measures and data collection techniques were approved by the group, who advocated the need for research into re-exacerbation and readmission prevention strategies in COPD.

## Results

**Figure 1:**
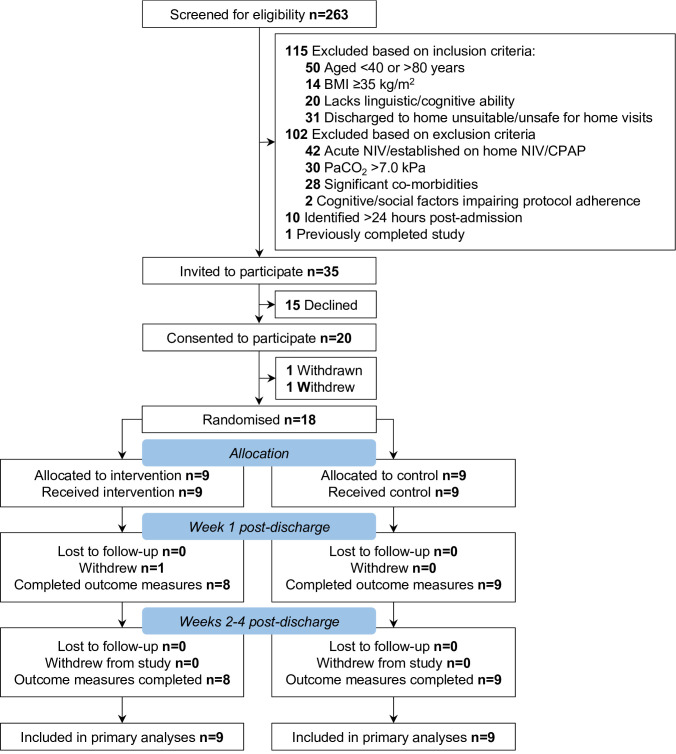
Participant flow diagram. BMI, body mass index; CPAP, continuous positive airways pressure; NIV, non-invasive ventilation; PaCO2, arterial partial pressure of carbon dioxide.

Recruitment was undertaken between June 2019 and March 2020 and terminated prematurely due to COVID-19 (figure 1). 263 patients were screened for trial participation with 34 patients (13%) fully meeting eligibility criteria. After consenting to participate and prior to randomisation, one patient was withdrawn due to the development of significant hypercapnia, and one patient withdrew following hospital self-discharge. Of 34 eligible patients, 18 were randomised. Participant characteristics are reported in table 1. All feasibility progression criteria were met: 18/34 (53%) were recruited and randomised, one (6%) withdrew after sustaining a pelvic fracture, ≥70% data were collected for each outcome measure (table 2) and there were no device-related SAEs.

**Table 1:** Participant characteristics at baseline assessment

	Total cohortn=18	Intervention armn=9	Control armn=9
Demographics and anthropometrics			
Age (years)	70±5.2	69±6.1	70±4.5
Female sex	8 (44)	2 (22)	6 (67)
Body mass index (kg/m^2^)	22.5±5.0	24.2±3.2	17.1 (16.7–27.3)
Lives alone	12 (67)	5 (56)	7 (78)
Smoking and exacerbation history			
Current smoker	8 (44)	3 (33)	5 (56)
Pack years	56±24	58±28	55±20
COPD severity			
Established on long-term oxygen therapy	1 (5)	0 (0)	1 (11)
Annual exacerbation frequency	4±3	4±3	3±2
Annual severe exacerbation frequency	1 (0–2)	1 (0–2)	0 (0–2)
FEV_1_ (L)*	0.71±0.28	0.85±0.36	0.60±0.16
FEV_1_ (% predicted)*	31.6±12.5	32.2±12.6	31±13.2
FEV_1_:FVC*	0.39±0.15	0.38±0.18	0.32±0.06
Comorbidities			
Ischaemic heart disease	1 (6)	0 (0)	1 (11)
Hypertension	6 (33)	4 (44)	2 (22)
Diabetes mellitus	3 (17)	2 (22)	1 (11)
Admission investigations			
Reported consolidation on admission chest radiograph	5 (28)	4 (44)	1 (11)
pH†	7.40±0.04	7.38±0.03	7.43±0.04
PaCO_2_ (kPa)†	5.48±0.56	5.75±0.34	5.18±0.64
PaO_2_ (kPa)†	8.21±1.68	8.60±1.68	7.76±1.72
HCO_3_^-^ (mmol/L)†	24.9±1.86	24.9±1.82	25.0±2.08
White cell count (×x10^9^/μL)	11.6±4.6	11.1±4.4	12.1±4.9
Eosinophils (×10^9^/μL)	0.1 (0.0–0.3)	0.2 (0.1–0.5)	0 (0–0.2)
CRP (mg/L)	15 (7–28)	10 (3–16)	23 (10–66)
Clinical outcomes			
Hospital length of stay	2 (1-2)	2 (1-2)	2 (2-3)
30-day non-readmission exacerbation	5 (28)	1 (11)	4 (44)
30-day readmission	3 (17)	2 (22)	1 (11)

Data presented as mean±SD, median (IQR) or n (%).

* Spirometry performed in 14 on admission (6six intervention arm, 8eight control arm).

† Arterial blood gas performed on admission in 13 patients (7seven intervention arm, 6six control arm),.

COPDchronic obstructive pulmonary diseaseCRPC-reactive proteinFEV_1_forced expiratory volume in 1 secondFVCforced vital capacityHCO_3_-bicarbonatePaCO_2_arterial partial pressure of carbon dioxide (CO_2_) or oxygen (O_2_)

**Table 2:** Completion of home-based outcome measures

Outcome measure	Total cohortn=18	Intervention armn=9	Control armn=9
Vital observations	17 (94%)	8 (89%)	9 (100%)
Questionnaires: CAT, CCQ, MDP	17 (94%)	8 (89%)	9 (100%)
Daily symptom diary: mBorg and VAS (days completed)	28 (19–30)	21 (17–29)	29 (22–30)
Spirometry	63 (98%)	31 (86%)	32 (100%)
Inspiratory capacity	67 (99%)	32 (89%)	35 (97%)
Parasternal electromyography	17 (94%)	8 (89%)	9 (100%)

CATCOPD assessment testCCQClinical COPD questionnaireCOPDchronic obstructive pulmonary disease mBorgmodified Borg scaleMDPmultidimensional dyspnoea profileVASvisual analogue scale

**Figure 2:**
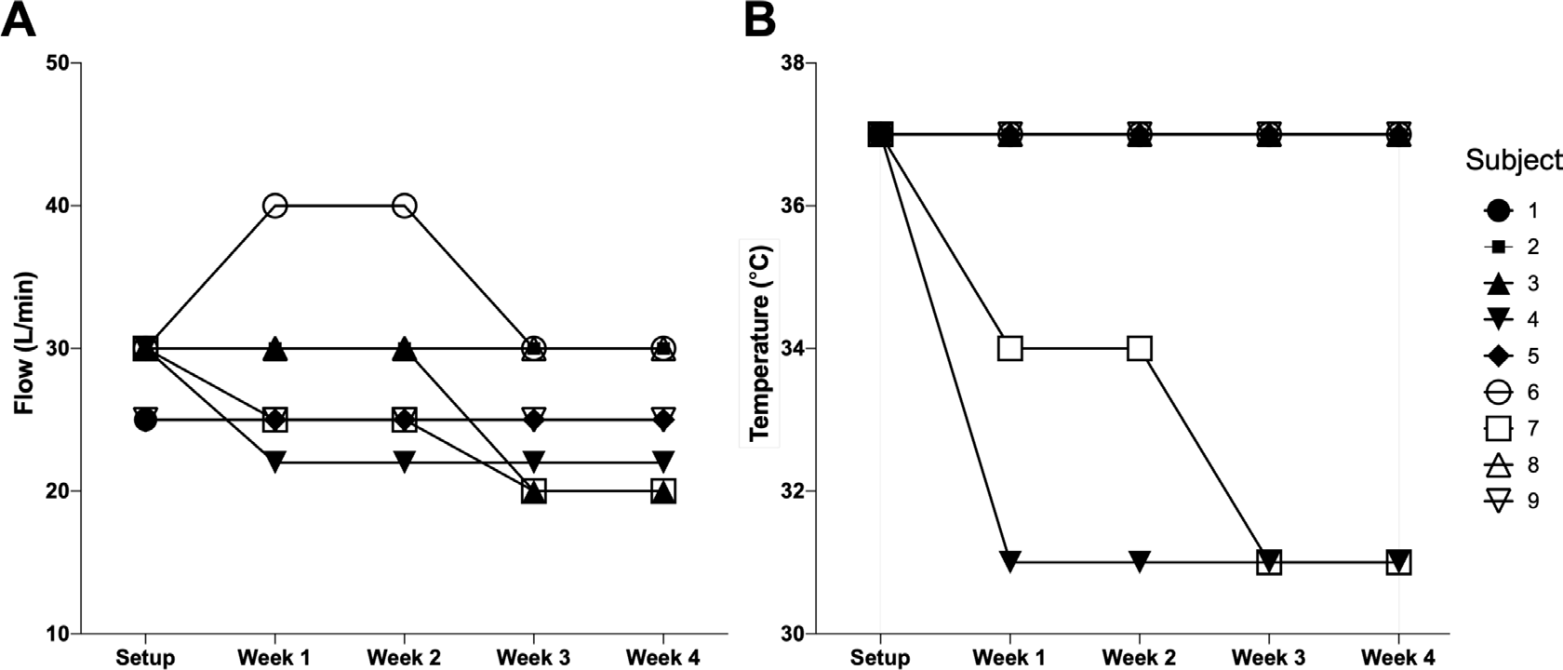
Home high-flow therapy settings during recovery from severe chronic obstructive pulmonary disease (COPD) exacerbation. (**A, B**) Temperature and flow settings, respectively, at setup and during follow-up.

**Figure 3:**
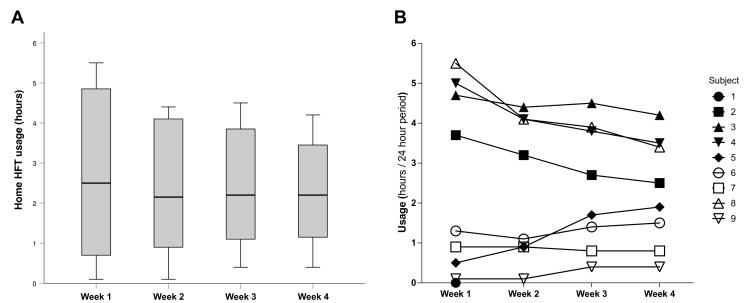
Home high-flow therapy usage in the 4 week postdischarge period following hospitalisation with severe COPD exacerbation. (**A**) A box plot depicting hours of device usage per 24-hour period for all subjects. (**B**) Individual usage per 24-hour period.

Regarding HFT settings on discharge following acclimatisation, all patients tolerated a temperature of 37°C, and 6/9 (67%) tolerated 30 L/min flow rate, with 3/9 (33%) accepting 25 L/min as the highest acceptable flow rate. Following hospital discharge, temperature was modified for two patients and flow titrated for four patients for comfort (figure 2). Home HFT usage per 24-hour period was 2.4±1.7 hours. Use was highest in week 1 post-discharge (2.7±2.2 hours), falling to 2.4±1.8 hours, 2.4±1.5 hours and 2.3±1.4 hours in weeks 2, 3 and 4, respectively (figure 3). No patients randomised to receive HFT required home oxygen and therefore oxygen entrainment for hypoxaemic respiratory failure.

There were six episodes of re-exacerbation in the study period, occurring in five patients, of which five were community-treated (four control, one intervention) and one required hospital readmission (control group). There were two readmissions for non-COPD-related reasons (pelvic fracture and left heart failure). 90-day mortality was 17% (one intervention, two control). Patient-reported and physiological outcomes are reported in table 3. The data show trends towards reduced symptom burden and improved physiological parameters (spirometric, neural drive, respiratory rate) over the study period. A weak dose-response relationship was observed for duration of daily home HFT use and total symptom scores (CAT r^2^ 0.20, p=0.01; CCQ r^2^ 0.24, p=0.01), cough (CAT r^2^ 0.26, p=0.002; CCQ r^2^ 0.18, p=0.03) and breathlessness (mBorg r^2^ 0.13, p=0.04). Actigraphy indices highlighted a trend to reduced immobile time in both arms during exacerbation recovery, with shorter sleep time and reduced sleep efficiency observed in participants using HFT.

**Table 3:** Patient reported and physiological outcomes during recovery from severe COPD exacerbation

Outcome	Group	Admission	Discharge	Week 1	Week 2	Week 3	Week 4
Patient-reported outcomes							
CAT total	HFT	33±4	23±7	23±5	23±7	21±6	20±6
	Control	31±5	26±7	23±8	22±7	20±7	19±8
CCQ total	HFT	39±9	30±14	30±9	28±10	29±8	28±11
	Control	40±7	31±6	29±12	26±11	26±8	27±10
CAT cough subdomain	HFT	4.5 (3–5)	2.5 (2–3)	2.5 (1–4)	2.5 (2–4)	2.5 (1–4)	2 (2–4)
	Control	4 (2.5–5)	4 (2.5–4.5)	3 (2-4)	2 (1.5–3.5)	2 (1.5–4)	2 (1.5–4)
CCQ cough subdomain	HFT	5 (2.5–5)	3 (2–4)	3 (2–3)	2.5 (1–4)	4 (2–4)	3 (2–4)
	Control	5 (4–6)	4 (3–5)	3 (3–4)	2 (2–4)	3 (2–3.5)	2 (2–4)
CAT sputum subdomain	HFT	4 (2–5)	2 (1–4)	3 (2-4)	2 (1–3)	2 (1–3)	2 (1–3)
	Control	4 (2.5–5)	4 (2–4.5)	2 (2–3.5)	3 (1–3.5)	2 (1–3.5)	2 (1–3.5)
CCQ sputum subdomain	HFT	3.5 (1-4)	2 (1–3.5)	2 (2–3)	2 (1–4)	2 (1–3)	2 (1–4)
	Control	3 (2–5)	2 (2–3.5)	2 (2–3)	2 (1.5–3.5)	3 (1.5–3.5)	2 (2–3.5)
mBorg for breathlessness	HFT	9.5 (6.5–10)	4.5 (3-6)	5 (4-5)	5 (4-7)	5 (3.5–6)	5 (3–5)
	Control	5 (4.5–9)	5 (3–5.5)	5 (3–5)	5 (3–6)	5 (2.5–5)	4.5 (2–7)
VAS for breathlessness	HFT	85 (66–98)	33 (23–48)	40 (33–42)	56 (42–72)	42 (24–54)	50 (37–51)
	Control	60 (45–85)	45 (40–50)	39 (32–52)	39 (28–59)	47 (28–55)	50 (20–58)
MDP sensory dimension	HFT	32±15	17±13	20±9	23±11	17±11	20±12
	Control	26±14	19±9	14±11	16±9	15±10	13±10
MDP affective dimension	HFT	37 (15–44)	16 (9–29)	15 (8–31)	13 (10–37)	10 (5–29)	23 (9–32)
	Control	22 (11–31)	13 (3–23)	13 (9–23)	11 (4–16)	8 (6–15)	8 (4–14)
Physiological outcomes							
Respiratory rate	HFT	25 (18–36)	20 (17–23)	20 (16–21)	21 (17–27)	20 (18–25)	19 (17–22)
	Control	22 (16–26)	16 (14–19)	17 (16–20)	19 (14–22)	18 (15–22)	19 (16–21)
SpO_2_ (%)	HFT	92 (90–96)	95 (91–95)	96 94–97)	96 (94–96)	95 (93–96)	95 (93–96)
	Control	93 (91–96)	92 (90–95)	94 (91–96)	96 (92–97)	95 (92–96)	96 (91–96)
FEV_1_ (L)	HFT	0.79 (0.52–1.23	0.76 (0.52–1.29)	0.76 (0.55–1.42)	0.79 (0.67–1.12)	0.78 (0.54–1.43)	0.81 (0.57–0.91)
	Control	0.60 (0.47–0.78)	0.62 (0.43–0.72)	0.72 (0.67–0.75)*	0.67 (0.54–0.93)	0.69 (0.64–0.85)*	0.63 (0.56–0.82)
FEV_1_ (% predicted)	HFT	32 (21–45)	29 (21–42)	30 (23–45)	29 (25–40)	29 (25–47)	28 (26–33)
	Control	26 (22–45)	25 (21–53)	33 (24–51)*	35 (25–47)	37 (23–51)*	31 (22–47)
FEV_1_:FVC	HFT	0.38 (0.35–0.70)	0.33 (0.30–0.45)	0.39 (0.33–0.58)	0.36 (0.33–0.63)	0.33 (0.31–0.57)	0.34 (0.31–0.51)
	Control	0.31 (0.27–0.38)	0.32 (0.28–0.36)	0.32 (0.31–0.34)	0.33 (0.32–0.34)	0.32 (0.28–0.35)	0.31 (0.29–0.36)
IC (L)	HFT	1.61±0.40	1.64±0.59	1.65±0.47	1.49±0.36	1.71±0.56	1.79±0.45
	Control	1.39±0.35	1.43±0.51	1.55±0.51	1.58±0.56	1.61±0.51	1.40±0.50
IC (% predicted)	HFT	55±10	55±19	56±13	52±13	59±18	61±12
	Control	63±22	69±22	70±27	75±28	74±28	64±28
EMG_para%max_ (%)	HFT	20±16	15±11	11±4	11±6	10±5	8±3
	Control	23±9	20±8	22±14	19±9	18±11	17±9
NRDI (%·bpm)	HFT	457±346	304±175	205±104	229±131	208±139	159±67
	Control	541±245	343±158	415±292	367±231	315±247	334±202
Immobile time (%)	HFT	–	–	30±11	34±9	27±8	26±9
	Control	–	–	30±9	32±12	29±12	27±10
TST (min)	HFT	–	–	341 (227–393)	332 (288–347)	249 (207–335)	262 (215–296)
	Control	–	–	422 (352–446)	384 (375–483)	424 (400–466)	409 (348–469)
Latency (min)	HFT	–	–	33 (18–80)	31 (18–62)	33 (15–70)	34 (17–63)
	Control	–	–	31 (13–64)	19 (6–53)	27 (10–70)	21 (13–48)
Sleep efficiency (%)	HFT	–	–	64 (45–65)	61 (55–64)	57 (41–60)	57 (39–65)
	Control	–	–	69 (66–79)	73 (68–80)	76 (67–81)	73 (69–78)

* Five intervention arm participants and six controls completed spirometry at each assessment. Data presented as mean ± standard deviation or median (interquartile range).

AC/minactivity count in arbitrary units per minuteCATCOPD Assessment TestCCQClinical COPD QuestionnaireCOPDchronic obstructive pulmonary diseaseEMG_para_parasternal electromyographyFEV_1_forced expiratory volume in 1 secondFVCforced vital capacityHFThigh-flow therapyICinspiratory capacitymBorgmodified Borg scaleMDPmultidimensional dyspnoea profileNRDIneural respiratory drive indexSpO_2_peripheral oxygen saturationTSTtotal sleep timeVASvisual analogue scale

### Qualitative data are presented below under Theoretical Framework of Acceptability (TFA) constructs

#### Affective attitude

Consistent with their willingness to participate in the trial, all patients expressed enthusiasm to try the novel intervention. Several described overall satisfaction: ‘I’m very pleased with it’ (P1) and ‘I think it’s a really great invention’ (P9). The interface was reportedly comfortable: ‘The nose part fits comfortable’ (P17) and ‘It’s relaxing, when you’re just lying there and it’s tickling your nose’ (P8). The sensation of heat was variably described as a facilitator ‘It just warms you up, it just makes you feel nice and warm’ (P10) and barrier (‘I found it too much when the heat was high’ (P13), which resolved with settings modification. HFT was perceived as promoting confidence in undertaking physical activities: ‘It’s given me more confidence in doing more about the house’ (P3) and a safety net (‘I think it would be very helpful on bad days, especially in winter’ (P10) and ‘Something to fall back on’ (P9)) in the event of clinical deterioration. One participant was worried by the potential for technical difficulties (‘It worried me…Here by myself, I felt that if something happen, then I be alone’ (P7)). Limescale build-up was noted in the humidification chamber, rectified after remembering to adhere to recommendations of using cooled boiled water.

#### Burden

HFT imposed no cognitive burdens and was reported as ‘easy’ (P3, P7 P10, P13, P18) and ‘straightforward*’* (P13, P17) to setup, use and maintain. No technical problems were encountered. Participants considered that the duration HFT could be worn may be limited by employment or adherence to additional medications and hospital appointments (‘On your working days you can’t and if you’re doing night work you can’t. I think it’s the amount of time you’ve got to spend on it every day which makes it a little bit awkward’ (P18), ‘I do like to do as many hours as I can, but you know there are other things you have to do, and plus all my pills I have to take and hospital appointments’ (P3)). The time taken for the device to heat to be ready to use was observed (‘It takes quite a while to heat up’ (P13)) but not described as a barrier: ‘It’s not as though I’m running a race, is it?…I don’t sit there and wait for it now. I just put it on, do a few other little things and then I think okay it’s ready now and then, you know, go’ (P3), ‘I switch my computer on and I let the dog out, I switch that on, I go and make my coffee, by the time I come back it’s ready to put on, so I’ve never timed it but it’s pretty quick’ (P17)).

#### Ethicality

The non-pharmacological nature of home HFT appealed: ‘I think why I used it so much and liked using it is because there’s no chemicals in it… I think that’s the main thing is that it hasn’t got any medication in it’ (P17) and ‘I think good idea because obviously it’s not chemical so the obvious thing which I mean it’s better for your lungs’ (P18). It also reminded one participant of steam inhalation home remedies: ‘My brother, he always says to me, stick your head over a bowl’ (P3).

#### Intervention coherence

The purpose of the device was clear to all participants: ‘I think it’s a good idea that machine to be quite honest…It makes a massive difference’ (P13), ‘The thing is, I think it’s doing me some good. I think it’s a really great invention’ (P9) and ‘It’s worth using as it feels good and makes you feel better’ (P10). Training was well received (‘The training I felt was spot on, it was very good…I didn’t have any problem using it after that’ (P3)). Having written information was felt to be beneficial (‘It’s difficult to remember everything, so it’s good have the leaflets so then you can always go back and look it up’ (P13). The warm-up time impeded the application of home HFT as an acute breathlessness relief tool, and it was thus viewed better suited as a maintenance therapy: ‘What I do is I use the shaker first and I put on the machine’ (P13).

#### Opportunity costs

Home HFT was uniformly incorporated into daytime routines, most commonly while watching television, sleeping, reading or working on the computer (‘I’ll sit there for a couple of hours when I’m watching my programmes on television because it doesn’t interfere with me at all’ (P3)). HFT was reportedly used at consistent times every day, with several patients specifying which hours they selected. Adherence to consistent times appeared to encourage regular use, meant that device usage did not impede on other day-to-day activities, and allowed patients to use it when they felt they would benefit most, for instance, following physical exertion: ‘So I go to the gym, do my exercises and whatever, and I’ll come back, then I’ll have a shower for myself. So, I’ll relax for half an hour, and I’ll use the machine’ (P13).

#### Perceived effectiveness

Home HFT was perceived as having demonstrable efficacy in improving symptoms, most notably sputum volume and clearance and also breathlessness. Patients noticed that after introduction of the device, their sputum was looser and easier to clear, lighter in colour and had reduced in volume: ‘I find now that I don’t get a quarter as much as I used to and I find it really has helped me in that respect, because it used to get stuck in my throat and it always made me feel sick. But since I’ve been using the device, it’s been a lot, lot better’ (P3), ‘I think it loosens it up and helps me bring it up quite easy’ (P9) and ‘Since I started using it, I wasn’t coughing up as much phlegm as I had been’ (P10). The impact of loosened secretions on cough was variable. Some patients observed that it increased cough, while others felt it was no different than following nebuliser use: ‘It obviously has that side effect of making you have to cough it all up afterwards…It loosens it off although you’ve got to keep blowing your nose at times’ (P17) and ‘I didn’t cough any more than I would cough using the nebuliser to be honest’ (P18). Home HFT influenced breathlessness in several patients, although feedback was less emphatic than for sputum production and clearance: ‘With the breathlessness, I’m not too sure. I think it did help’ (P10), ‘Well, it’s helped with my breathing I think, I think it’s helped with my breathing. I think that’s what it is’ (P9) and ‘Normally when I’m walking about for a little while, I’m trying to fight for my breath but although I do still, not half as much as I did so no, it’s been very beneficial’ (P3). Patients reported a reduction in use of as-required inhaled bronchodilation: ‘To be honest I haven’t been using (salbutamol) much at all…sometimes I’d use it maybe three or four times a day. But thinking about it now, that’s the first time I’ve used the blue inhaler since I saw you last week’ (P17) and ‘Obviously you don’t use your puffer so much’ (P18). Few patients observed a change in functional ability following introduction of home HFT: ‘I’d say that I can walk a bit further than I used to let’s put it that way, yeah’ (P6) and ‘I didn’t think (climbing stairs) was any different using that’ (P2).

#### Self-efficacy

Patients were confident and empowered to setup and use home HFT immediately following hospital discharge. One patient remarked that he found it difficult to decide whether to use his nebuliser or HFT. Another patient who lived alone, despite early enthusiasm, technical capability and high usage, described progressively deteriorating confidence in home HFT use. He ascribed this to the inability to rapidly access support in the event of clinical or technical difficulties, unlike in the hospital setting. Conversely, an older male who had close support from his daughter developed improved confidence and autonomy following hospital discharge: ‘I told myself well my daughter’s not going to be here all the time, so I’ve got to do things for myself and that’s what I did. Yeah, and then I wasn’t too sure, but I’ve fathomed it out. I’ve worked it out and it was easy, really’ (P9).

Quantitative and qualitative integration within a convergence coding matrix is presented in table 4.

**Table 4:** Convergence coding matrix integrating quantitative and qualitative datasets to determine the feasibility and acceptability of home high-flow therapy during severe COPD exacerbation recovery

Meta-theme	Quantitative	Qualitative	Convergence assessment
Clinical outcomes			
Re-exacerbation and readmission risk	There were six re-exacerbation/readmission events occurring in five patients. Five were community treated (four control, one intervention); there was one readmission (control).	Re-exacerbations, including use of rescue packs or hospital admissions, were not described.	*Silence*: re-exacerbations or hospital readmissions were not discussed at interview.
Use of prescribed bronchodilation	Use of ‘as-required’ inhaled or nebulised salbutamol was not quantified.	Patients reported reduced use of their ‘as required’ bronchodilation following introduction of home HFT.	*Silence*: salbutamol use was not objectively quantified. Patients expressed a preference for reducing medication usage and the non-pharmacological appeal of HFT.
Safety	No device-related adverse events were reported.	The device was well tolerated with no significant side effects impeding its application. Increased nasal discharge was reported by one patient. Temperature and flow settings were titrated to comfort in four patients.	*Agreement*: HFT is a safe device to use in the home setting.
Acceptability			
Recruitment and attrition	Progression criteria were met: 40% eligible patients were recruited, and 15% lost to follow-up. Completion of outcome measures was 81–100% and was lowest for spirometry.	Consenting patients were interested in using the device, understood its purpose and were enthusiastic to try it. The patients who withdrew or were withdrawn following randomisation did so for non-respiratory acute clinical reasons.	*Agreement*: recruitment and attrition rates were consistent with other randomised clinical trials of medical devices, with consenting patients demonstrating motivation to engage with a novel therapy.
Home HFT usage pattern	Average device usage was 2.4 hours per 24-hour period. Usage was highest in week 1 and progressively reduced during follow-up. Dose-response correlations were observed between hours of use and breathlessness, cough and overall COPD symptom burden. Flow settings were modified in four patients for comfort.	Patients understood the purpose of the device and its mechanisms of action (heating and humidification). Home HFT was easy to operate, no technical difficulties were encountered. No opportunity costs were reported with home HFT rapidly incorporated into daily routines. Perceived symptomatic benefits encouraged HFT use. Temperature and flow could be uncomfortable, but resolved after modifying settings.	*Agreement*: regular daily use was observed. Tangible perceived effectiveness, the comfortable nature of the intervention and minimal opportunity costs may have contributed to regular usage. The trend to a reduction in daily use may relate to improving symptoms and the perceived need for HFT usage falling. Patients demonstrated intervention coherence and self-efficacy.
HFT burden and affective attitude	Health related quality of life, as measured with the CAT and CCQ questionnaires, improved in both the intervention and control arms, with higher scores consistently observed at home-based follow-up in the intervention arm. Sensory and affective dimensions of MDP scores were higher in home HFT users.	Initial scepticism about potential effect of home HFT was reported, as were psychological barriers inhibiting its use, relating to lack of home-based monitoring and access to support. Stigma and fear of worrying family members was described. The sensation of heat and flow was comfortable and relaxing; however, no-one reported prolonged overnight or daytime use. The device was reassuring for some and gave them confidence and a sense of independence. The lack of chemicals was a positive. Overall satisfaction was conveyed. Start-up time was a burden for some. There was no cognitive burden associated with HFT since it was uniformly reported as easy to use, and training was considered to be valuable and of good quality.	*Partial agreement*: quantitative measures of HRQoL improved; however, sensory-affective dimensions of breathlessness scores were static or deteriorated, which may relate to psychological influences. Qualitative reports of device-related scepticism, anxiety and stigma, and burdens of regular use were divergent with CAT and CCQ scores and convergent with MDP sensory-affective scores. General satisfaction, low cognitive and time burdens and the sense of reassurance associated with home HFT were convergent with CAT and CCQ scores. Home HFT demonstrated ethicality, with its non-pharmacological nature appealing.
Patient-reported outcomes			
Cough and sputum	There were no gross differences in cough scores (CAT or CCQ subdomains) following randomisation to HFT.	The main symptomatic benefit reported by patients was reduction in sputum volume and improved clearance. Increased nasal discharge was reported and felt to be tolerable and expected.	*Disagreement*: sputum clearance was emphatically described as the most beneficial symptomatic effect of home HFT, which was not captured by questionnaires.
Breathlessness	Following discharge, breathlessness improved in both arms. Scores were more variable in the intervention arm, compared with stable scores observed in the control arm. Sensory and affective dimensions of breathlessness scores were higher in home HFT users.	Improvements in breathlessness were reported, although less emphatically than sputum clearance Three patients reported a noticeable reduction in their use of ‘as required’ inhaled or nebulised bronchodilators.	*Agreement*: breathlessness improved among home HFT users, although these benefits were not as pronounced as for sputum clearance.
Physical activity and sleep	Daytime physical activity fluctuated during home-based follow-up, and a trend to reduced proportion of daytime spent immobile was observed. Total sleep time and sleep efficiency decreased among home HFT users.	Ability to perform daytime activity was not commonly reported, with conflicting reports of increased functional capacity compared with no observed changes. No patients described sleep quality or sleep disruption, although discomfort and fear relating to nasal discharge and sputum clearance while in the supine position was reported.	*Agreement*: there was no clear influence of home HFT use on daytime physical activity.*Silence*: changes in sleep quality were not described qualitatively; however, reported positional changes in nasal and chest clearance may be a barrier to overnight use.
Physiological outcomes			
Spirometry and inspiratory capacity	There was a trend to reduced hyperinflation, as demonstrated by increasing forced vital capacity and inspiratory capacity during study participation.	Lung volumes were not discussed.	*Silence*: measures of expiratory airflow and hyperinflation were not discussed at interview.
Neural respiratory drive	There was a steeper trajectory of neural respiratory drive improvement (EMG_para_, EMG_para%max_ and NRDI) following randomisation to home HFT that was not observed in control arm participants.	The effort of breathing and sensation of breathlessness was reported to have improved following home HFT introduction.	*Agreement*: improvements in neural respiratory drive were perceived as a reduction in the effort of breathing and sensation of breathlessness among home HFT users.

CATCOPD assessment testCCQClinical COPD QuestionnaireCOPDchronic obstructive pulmonary diseaseEMG_para_mean parasternal electromyographyHFThigh-flow therapyMDPmultidimensional dyspnoea profileNRDIneural respiratory drive index

## Discussion

This is the first study to evaluate the feasibility of randomising eucapnic patients recovering from severe COPD exacerbation to home high-flow therapy prior to hospital discharge. All progression criteria were met, demonstrating that this trial design is feasible with acceptable recruitment proportions, satisfactory outcome data capture and no concerning device-related adverse effects. Furthermore, the quantitative and qualitative data support home HFT use during severe COPD exacerbation recovery as an acceptable intervention to patients.

### Feasibility

The primary outcome of this study was protocol feasibility. Recruitment and randomisation was consistent with comparable RCTs of respiratory support devices in COPD.^8 16^ Attrition was low and related to non-respiratory acute events. Completion of outcome measures was high, with an expected lower ability to perform technically acceptable spirometry during a severe COPD exacerbation. Patient-reported benefits, notably sputum clearance and breathlessness, were identified using quantitative questionnaire and qualitative interviews. No device-related adverse events were reported, consistent with the reported safety profile of home HFT.^16 17^

### High-flow therapy (HFT) setup and adherence

Device-related discomfort was reported by four patients at follow-up, with prompt resolution (within seconds to minutes) of actively titrating temperature or flow to comfort. This is consistent with the findings of Roca *et al*, who observed immediate resolution of discomfort following flow reduction in patients with acute respiratory failure.^39^ HFT usage per 24-hour period in this study was higher than patients with stable COPD or bronchiectasis evaluated by Rea *et al* (mean 1.6 hours/24-hour period) and comparable to bronchiectasis patients studied by Hasani *et al* (3 hours/day), in whom heated humidification conferred mucociliary clearance, time to first exacerbation and HRQoL benefits.^40 41^ Adherence was lower than in stable COPD patients with hypoxaemic respiratory failure studied by Storgaard *et al* (mean 6 hours/24-hour period) and stable COPD patients with or without hypoxaemia recruited within 3 months of hospitalisation with exacerbation evaluated by Criner *et al* (mean 6.8 hours/24-hour period).^16 42^ It is conceivable that the presented cohort, who were naïve to respiratory support therapies (including LTOT or NIV), were unaccustomed to sustained use of a novel medical device. This highlights the need for greater education and support to be provided as part of a phase 3 trial. Home HFT adherence reduced during COPD exacerbation recovery, indicating that patients perceived less benefits from using home HFT with symptom resolution. Consideration of flow and pressure titration to comfort should be given in a future trial.

### Patient-reported outcomes

Sputum production and clearance were emphatically reported by patients at interview as the most important effect of home HFT use. Heated humidification to 37°C 100% relative humidity has been established in animal models to optimise respiratory epithelial function, including ciliary beat frequency and mucus transport velocity.^43^ This was applied in a clinical trial undertaken by Hasani *et al* who demonstrated enhanced clearance of respiratory secretions by patients with bronchiectasis following consistent daily HFT use.^41^ The qualitative findings of the current study are consistent with previous reports, in which stable hypoxaemic patients describe reduced sputum volume and tenacity with regular home HFT use.^44^ Sputum clearance should be evaluated in a future clinical trial; however, this study highlights important limitations of existing quantitative questionnaires used to do so. Alternative approaches could include a visual analogue scale or numerical rating scale for sputum clearance.^45^

While this study was not powered to detect clinical change, the impact of home HFT on respiratory symptoms and HRQoL in this dataset is similar to published reports from stable and acutely hypoxaemic COPD patients.^16 46^ Reduced short-acting beta-agonist usage was qualitatively reported and should be quantified in a future trial.

### Chronic obstructive pulmonary disease (COPD) re-exacerbation and readmission

COPD re-exacerbation and hospital readmission rates in this study are consistent with national and international data.^2 47^ RCT data in patients with stable COPD and respiratory failure have demonstrated that application of home HFT is associated with reduced risk of exacerbation over 12 months. However, none of these studies have demonstrated an effect on hospital admission rate.^16 17^ Temporal clustering of COPD exacerbations has been described, with a high-risk period for recurrent exacerbations reported in the 2 months following the index exacerbation,^48^ with 15% of eucapnic patients readmitted within 14 days and up to 24% within 30 days^1 26^. Therefore, if an intervention can be demonstrated to interrupt this cycle, exacerbation frequency may improve, and long-term data are warranted.

### Study limitations

This trial was truncated due to the onset of the COVID-19 pandemic which rendered recruitment unfeasible due study team redeployment and reduced AECOPD admissions.^49^ However, participant numbers were considered sufficient to determine the primary outcome of protocol feasibility, based on methodological guidance on sample sizes for feasibility trials.^50^ A strength of this study was application of mixed methodology. This facilitated identification of the most tangible benefit of home HFT for patients, which was sputum production and clearance. This observation was not captured in the quantitative questionnaires implemented. Furthermore, conducting home-based rather than hospital/laboratory based assessments, which carry significant patient burden,^51^ facilitated adequate recruitment, retention and data collection.

## Conclusion

This mixed methods feasibility randomised controlled trial determined that it is feasible to conduct a Phase 3 clinical trial to investigate the effects of home high-flow therapy in eucapnic COPD patients recovering from a severe exacerbation, as evidenced by the recruitment and retention, data collection, device safety and acceptability. Home high-flow therapy applied in this context is safe and acceptable to patients and may confer symptomatic and physiological benefits. Quantifying sputum clearance and short-acting beta-agonist usage were identified as valuable outcome measures to be included in the phase three clinical trial. With feasibility demonstrated, an RCT to investigate whether home HFT, in addition to standard care, increases 12-month admission-free survival following severe COPD exacerbation is currently recruiting (ISRCTN89405844).

### Take home message

It is feasible to conduct a clinical trial to evaluate whether home high-flow therapy (HFT) can reduce re-exacerbations and readmissions during severe COPD exacerbation recovery. Home HFT is safe and may improve sputum clearance and reduce salbutamol use.

## Data Availability

All data relevant to the study are included in the article.
